# Decreased levels of metalloproteinase-9 and angiogenic factors in skin lesions of patients with psoriatic arthritis after therapy with anti-TNF-α

**DOI:** 10.1186/1740-2557-3-5

**Published:** 2006-10-05

**Authors:** Paola Cordiali-Fei, Elisabetta Trento, Giovanna D'Agosto, Valentina Bordignon, Anna Mussi, Marco Ardigò, Antonio Mastroianni, Antonella Vento, Francesco Solivetti, Enzo Berardesca, Fabrizio Ensoli

**Affiliations:** 1 Clinical Pathology and Microbiology Laboratory, San Gallicano Dermatology Institute, 00144 Rome, Italy; 2 Clinical Dermatology Division, San Gallicano Dermatology Institute, 00144 Rome, Italy; 3 Radiology Division – San Gallicano Dermatology Institute, 00144 Rome, Italy

## Abstract

**Background:**

Inflammation represents an early and key event in the development of both the cutaneous psoriasis and psoriatic arthritis. Compelling evidences indicate that the production of TNF-α plays a central role in psoriasis by sustaining the inflammatory process in the skin as well as in the joints. Among the multiple effects produced by TNF-α on keratinocytes, the induction of matrix metalloproteinase-9 (MMP-9), a collagenase implicated in joint inflammatory arthritis which acts as an angiogenesis promoting factor, might represent a key mechanism in the pathogenesis of the disease. Aims of the present study were to investigate a) the role of MMP-9 in the development of psoriasis by assessing the presence of MMP-9 in lesional skin and in sera of psoriatic patients; b) the association of MMP-9 with the activity of the disease; c) the relationship between MMP-9 and TNF-α production.

**Methods:**

Eleven psoriatic patients, clinically presenting joint symptoms associated to the cutaneous disease, were included in a therapeutic protocol based on the administration of anti-TNF-α monoclonal antibody (Infliximab). Sera and skin biopsies were collected before treatment and after 6 weeks of therapy. Tissues were kept in short term cultures and production soluble mediators such as TNF-α, MMP-9, MMP-2, VEGF and E-Selectin, which include angiogenic molecules associated to the development of plaque psoriasis, were measured in the culture supernatants by immunoenzymatic assays (ng/ml or pg/ml per mg of tissue). MMP-9 concentrations were also measured in the sera. The cutaneous activity of disease was evaluated by the Psoriasis Area and Severity Index (PASI).

**Results:**

Clinical and laboratory assessment indicated that all but one patients had a significant improvement of the PASI score after three months of therapy. The clinical amelioration was associated to a significant decrease of MMP-9 (P = 0.017), TNF-α (P = 0.005) and E-selectin (P = 0.018) levels, spontaneously released by lesional biopsies before and after therapy. In addition, significant correlations were found between the PASI measurements and TNF-α (r^2 ^= 0.33, P = 0.005), MMP-9 (r^2 ^= 0.25, P = 0.017), E-selectin (r^2 ^= 0.24, P = 0.018) production. MMP-9 levels were significantly correlated with those of TNF-α (r^2 ^= 0.30, P = 0.008). A significant decrease of MMP-9 in the sera, associated to the clinical improvement was also found.

**Conclusion:**

Our findings show the existence of a direct relationship between MMP-9 and TNF-α production strongly suggesting that MMP-9 may play a key role in the skin inflammatory process in psoriasis.

## Background

Psoriasis is a chronic inflammatory skin disease characterized by hyperproliferation of epidermal cells with prominent blood vessels and a thick perivascular lymphocytic infiltrate [1]. The etiology of psoriasis is still unknown. Whether the disease results from a primary abnormality in epidermal keratinocytes or depends upon a deregulation of the immune system it has been matter of controversial debating [2]. Recent evidence, however, indicated that activated lymphocytes and keratinocytes are both required for the development of psoriatic lesion [3]. In fact, the chronic, self-aggressive, epidermal T cells activation, characteristic of the disease, might be initiated by common streptococcal infections (β-haemolitic streptococci) that, in turn, might trigger a cross immune recognition between streptococcal M proteins and those keratins that are pathologically up-regulated in psoriatic lesions [4]. An auto-immune response may thus be sustained by a mechanism of molecular mimicry. Indeed, psoriatic keratinocytes exhibit an altered phenotype characterized by a costitutive Stat3 activation [3] and a different response to IFN-γ compared to normal keratinocytes [5,6].

About one third of patients with psoriasis also suffer of an inflammatory arthritis with clinical and biological features that are partially similar to rheumatoid arthritis (RA) [7]. Although, the systematic classification and the diagnostic criteria for this form of arthritis are still under validation [8], psoriatic arthritis (PsA) has been recognized as a clinical entity distinct from RA, due to the absence of the rheumatoid factor as well to the presence of specific clinical features. Articular erosions in PsA occurr less commonly than in RA and progression to joint destruction occurs at a slower rate, nevertheless it can lead to disability [9].

Compelling evidences indicate that TNF-α plays a central role in sustaining the psoriatic inflammatory process in skin as well as in joints [9,10]. In psoriatic skin, TNF-α is the prevalent cytokine: it can be produced by several cell types including activated T cells, keratinocytes, macrophages and Langerhans cells [11]. Moreover, in epidermal keratinocytes, TNF-α regulates genes involved in immune and inflammatory response, including those involved in cell motility or cytoskeleton changes or in extracellular matrix remodelling [12]. In the affected joints TNF-α appears as well responsible of the positive regulation and overexpression of chemokines, cytokines and angiogenic molecules which may lead to proliferation and activation of sinovial cells that, in turn, lead to cartilage and bone destruction [13]. Moreover TNF-α has been implicated in promoting osteoclastogenesis in PsA [14].

Therapeutic approaches based on anti-TNF-α agents have provided indirect evidence in support to this hypothesis, since they are highly effective in controlling both skin and joint manifestations in patients with PsA [15,16]. We have recently demonstrated that the marked improvement in both skin and joint manifestations following therapy with Infliximab, a chimeric monoclonal antibody which binds specifically to human TNF-α [17], was significantly associated to the decrease of serum levels of TNF-α, angiogenic molecules and MMP-2 [18]. MMPs belong to a family of proteolytic enzymes that are capable of degrading all components of the extracellular matrix, a key event in the development of cartilage destruction and joint erosion [19]. Kinetic studies using known model substrates have shown that both MMP2 and MMP9 are most effective in degrading collagen as compared to other MMPs [20] and have been specifically implicated in inflammatory arthritis, including angiogenesis [21-23]. In a recent paper we described significant decreases of IL-6, VEGF, FGF-b and E-selectin after early infliximab infusions and the significant correlations between PASI and MMP-2, FGF-b or VEGF [18].

In the present study we investigated the modifications of TNF-α, MMP-9 and MMP-2 released by the cellular elements located in the skin lesional tissue of patients with PsA, during treatment with Infliximab. We also determined the amounts of endothelial activation VEGF and E-selectin molecules, which are significantly associated to disease activity in psoriasis plaques [24,25]. MMP-9 levels were also determined in the sera.

The results showed a significant decrease of lesional amounts of MMP-2, MMP-9, TNF-α and E-selectin, in association with the clinical improvement of disease symptoms, occurring after three weeks of Infliximab monotherapy. A significant correlation was found also between peripheral levels of MMP-9 and PASI, differently from what previously found analysing MMP-2 serum levels [18].

## Patients and methods

### Patients

Eleven patients (9 males, 2 females) aged 35–60 years with a clinical diagnosis of PsA were included in the study upon releasing of a written informed consent. They were part of a larger group of patients partecipating to a monotherapy therapeutic protocol based on Infliximab administration. The study protocol was approved by the Hospital Ethics Committee. The design of the study and the enrollement criteria have already been described [18]. Briefly, Infliximab (5 mg/kg) was administered by intravenous infusion at week 0 and at weeks 2 and 6, and subsequently every 12 weeks. Disease activity was monitored at baseline, and before each programmed infusion. Skin involvement was evaluated with Psoriasis Area and Severity Index (PASI) [26]. Arthritis was evaluated following the American College of Rheumatology criteria (ACR) [27] with clinical examination of the tender and swollen joints, laboratory tests of inflammatory markers (erythrocyte sedimentation rate = ESR and C-reactive protein = CRP) and radiological examination of joint damage. An effective response to therapy was classified on the basis of improvement of PASI and/or ACR greater than 50% in comparison with baseline values.

### Short-term organ cultures

Lesional tissue specimens (4 mm punch biopsies) were taken at the enrollement visit and at week 6, before the third drug infusion, in the same skin area. Each tissue sample was rinced in cold sterile medium, weighed after removing fluid excess, and kept in short term culture in a polypropilene tube with 1 ml of complete medium (RPMI 1640, 10% FCS, 2 mM L-glutamine, antibiotics) in a 5% CO2 atmosphere at 37°C. After 36 hrs of incubation, the supernatant was collected, spinned in a cold microcentrifuge at 1000 × g and subdivided in small aliquots to be stored frozen at -80°C, until testing. After culture, biopsies were frozen and cryostatic tissue sections were examined: the structure of the skin was well preserved and included the dermis up to the reticular region.

### Sera

Blood samples were collected before drug infusion at week 0 and week 6. Sera were stored frozen in small aliquots for cytokine assays.

### Cytokine assays

The amounts of different biological mediators (TNF-α, VEGF, E-selectin, MMP-2, MMP-9) released in culture supernatants from the lesional tissue samples were measured by quantitative enzyme immunoassays (Quantikine Immunoassays, R&D Systems, Minneapolis, MN, USA), following the manufacturer's instructions. Samples and standards were analyzed in duplicate. Results were expressed as pg/ml or ng/ml per milligram of tissue. The sensitivity of each assay, according to the manufacturer, are reported in Table 1. MMP-9 amounts were additionally measured in the sera of patients collected concomitantly to skin biopsies.

**Table 1:** Improvement of psoriasis cutaneous expression after therapyand MMPs or cytokine levels released in tissue culture supernantans

	**Baseline values**	**After 6 weeks**	**Paired t P**
**PASI score**	19.1 ± 7.8	7.4 ± 2.7	0.0002
TNF-α *(pg/ml) **0.12	294.7 ± 123.5	194.9 ± 67.8	0.02
MMP-9 *(ng/ml) **0.15	207.3 ± 240.6	43.3 ± 73.9	0.03
MMP-2 *(ng/ml) **0.16	1404.0 ± 1081.0	1205.0 ± 1142.0	0.048
VEGF *(pg/ml) **9.0	510.7 ± 376.6	240.6 ± 270.1	0.02
E-Selectin *(ng/ml) **0.1	18.9 ± 23.5	4.1 ± 3.3	0.05

*Data are normalized per mg of tissue – **minimum detectable dose according to the manufacturer

### Statistical analysis

Cytokine measurements released before treatment were compared with those determined after therapy, by the paired t test, for the group of variables showed a normal distribution by the Kolmogorov-Smirnov (KS) test. Differences were considered significant when a two tailed P < 0.05 was obtained. The correlation between the PASI scores and the lesional levels of cytokines and MMPs and was evaluated by the Pearson correlation coefficient r^2 ^and two-tailed P value. Statistical analysis was performed by using the Graph Pad Software Prism 4 (GraphPad Software Inc., San Diego, CA).

## Results and discussion

### Clinical evaluation

Biological therapy based on monoclonal antibodies against TNF-α has been proven to be effective in patients with psoriatic arthritis on both the arthropaty and the cutaneous symptoms of the disease [15,16,28]. Today there are three main biological agents targeting TNF-α, which are already in use for treating PsA. These are the chimeric monoclonal IgG1 antibody infliximab with human constant and murine variable regions, the fully human anti-TN F-α monoclonal antibody adalimumab and the recombinant 75-kDa TNF receptor IgG1 fusion protein etanercept [29]. All catch soluble TNF-α in the plasma, only etanercept also bind to TNF-β. Infliximab and adalimumab also bind to cell membrane bound TNF-α, which may lead to cell lysis. There are several reports on the efficacy of infliximab in PsA [16,18]. Recent data from large placebo-controlled studies have shown that the medication is highly effective [30,31].

In the present study a group of patients with PsA, which underwent a monotherapy with Infliximab, also showed clinical improvements in skin and joint disease, as referred in a previous study [18]. At week 6 the ACR indexes showed a 50% improvement as compared to baseline values (week 0) in all patients. The PASI score at baseline ranged between 8.9 and 30.2, mean value: 19.06 ± 7.78; at week 6, it ranged between 4.5 and 12.3, mean value: 7.4 ± 2.68 (P = 0.0002). Ten patients showed an improvement >= 50% of the skin lesions (mean value of PASI improvement: 62.65 % ± 8.95 SD). One patient, with mild skin disease (baseline PASI score = 10.1), did not show a significant improvement after 6 weeks (PASI score = 10.0). Data are described in detail in Table 1.

### Cytokine levels

In the present study we found that the clinical improvement of the skin expression of the disease in patients with psoriatic arthritis under Infliximab significantly correlated with the decrease of lesional MMP-9 in association with the decrease of TNF-α, or VEGF and E-selectin, bioactive molecules already known to be implicated in the pathogenesis and clinical activity of the disease [32]. The approach used in the present study to measure the lesional levels of soluble molecules has been already adopted in previous studies demonstrative of the presence of inflammatory cytokines in psoriatic lesional tissue in comparison to uninvolved as well as normal skin samples [33,34].

**Figure 1:**
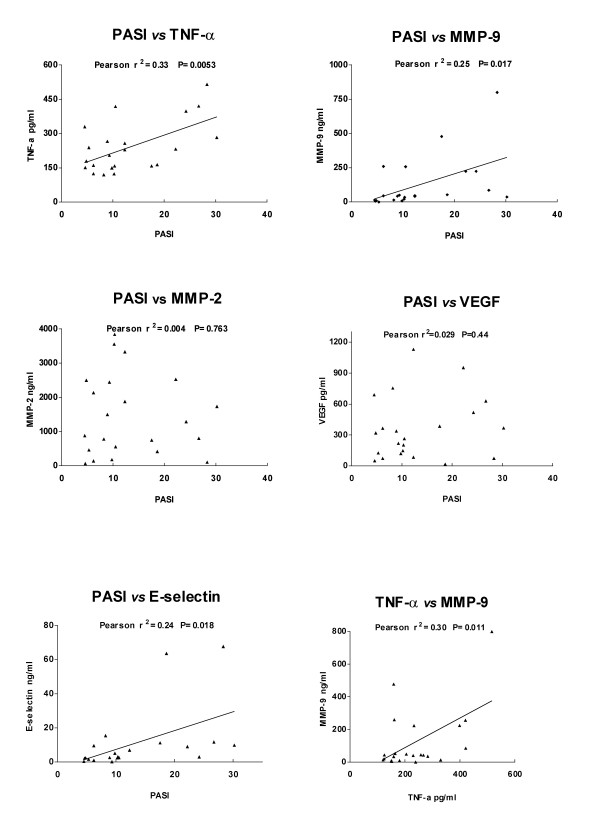
Correlation plots and linear regression lines between cutaneous expression of disease (PASI score) and TNF-α, MMP-9, VEGF, MMP-2, E-selectin amounts released by lesional tissue in short term cultures. The correlation and linear regression curve between TNF-α and MMP-9 is also shown.

By using this methodology, the mean values of TNF-α, VEGF, E-selectin, MMP-2 and MMP-9 released by lesional tissues before or after therapy have been measured and the results are summarized in Table 1. A significant decrease of all molecules was found after therapy. To establish a quantifiable relationship between the severity of cutaneous disease expression and the lesional levels of TNF-α, VEGF, E-selectin, MMP-2 or MMP-9, the correlation coefficients of each cytokine and the PASI score, taken at the same time as the corresponding of lesional biopsy, were calculated by the Pearson correlation test. The diagram in Fig. 1 depicts the plotted results of correlation and linear regression analysis. A statistically significant correlation was found between the PASI score and TNF-α, MMP-9 or E-selectin concentrations. Expression of TNF-α and MMP-9 were also significantly correlated.

TNF-α is considered a key cytokine regulator in psoriasis [35]. As expected, the lesional amounts of TNF-α were directly correlated with the PASI score, sustaining its pathogenic role in the development of skin psoriasis. The correlation found between MMP-9 and PASI, suggests that also this molecule may be involved in the inflammatory process leading to the cutaneous lesions. Indeed, TNF-α acts as a potent inducer of MMP-9 in keratinocytes [12]. Therefore, TNF-α mediated induction of MMP-9 could be responsible of the overexpression and activity of this molecule as found in the synovial cells and skin of patients with PsA [36-38,22]. On the other hand, MMP-9 can be also secreted by inflammatory cells, such as neutrophils, upon activation by the IL-8 family proteins [39]. Moreover, MMP-9 mediates terminal cleavage of IL-8, thus potentiating IL-8-induced activation of neutrophils [40]. This suggest that MMP-9 can be involved as a mediator of the IL-8-induced inflammatory process in the psoriatic skin [41]. The use of supernatants from short-term cultured punch biopsies cannot allow to define the target cells producing MMP-9. However, recent data obtained by immunohistochemical analysis of psoriatic skin and synovia in individuals under infliximab treatment showed that the drug decreased the neoangiogenesis and reduced the activation of the endothelial cells resulting in decreased cell infiltration and clinical improvement [42].

**Figure 2:**
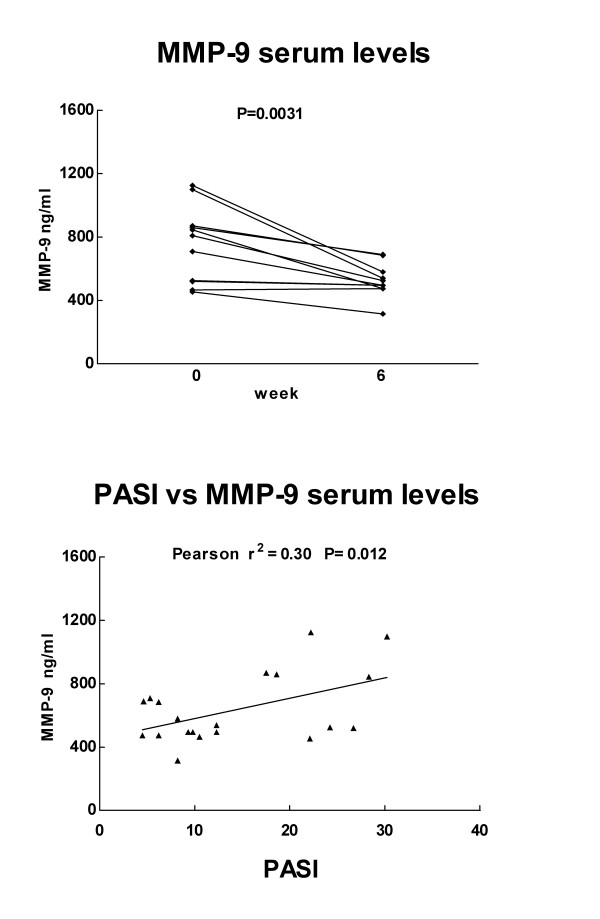
a) Comparison between MMP-9 levels (ng/ml), measured in the sera of 11 patients, before and after 6 weeks of Infliximab monotherapy; b) correlation plot and linear regression line between overall cutaneous expression of disease (PASI score) and MMP-9 serum levels.

Differently from that found in the sera [18], MMP-2 amounts in the skin lesions were scarcely modified by Infliximab monotherapy (P = 0.048), while MMP-9 lesional and serum levels were both strongly reduced. MMP-9 levels found in the sera collected before the third drug infusion were significantly reduced as compared to baseline values (Fig. 2a). Moreover, serum MMP-9 and TNF-α levels were significantly correlated as shown in the plot depicted in of Fig. 2b, referring to cumulative measures. Although the correlation coefficient values are low, the statistical significance in complex primary culture model system such as the skin and in the relatively small-sized population sample studied here suggest that the observed correlations might be of biological importance.

This finding might suggest that MMP-2, a type IV collagenase secreted by fibroblasts, is mainly involved in joint lesions of PsA, while MMP-9 contributes to the chronic inflammatory process in the skin of the psoriatic patients either directly, by sustaining the inflammatory process and the tissue distruction or indirectly, by allowing the traffic of inflammatory cells and enhancing the activity of inflammatory cytokines. In fact, experimental evidences indicate that MMPs can mediate the proteolytic process leading to the release of the soluble, active molecule of TNF-α from a cell membrane-achored molecular form [43]. In addition, due to its proteolytic activity, MMP-9 could contribute to the generation of immunogenic fragments of normal proteins that may offer the basis for the initiation of local autoimmune cellular responses.

The inhibitory effect of anti- TNF-α therapy may thus offer a two-fold efficacy through the reduction of MMP-9 levels and the inhibition of processing of TNF-α precursor into its active molecular form.

A knowledge of the fine mechanism regulating soluble factors such as the MMPs, which also have a key role in tissue repair [44], can help understanding the pathogenic mechanisms underlying the generation of psoriatic lesions and may thus have important therapeutic implications representing a powerful tool with which to direct new therapeutic strategies.

## Abbreviations

PsA: Psoriatic Arthritis

RA: Rheumatoid Arthritis

MMPs: Matrix Metalloproteinases

PASI : Psoriasis Area and Severity Index

TNF-α : Tumor Necrosis Factor-α

VEGF: Vascular Endothelial Growth Factor
